# The Body as a Vessel for Trauma: The Clinical Case Study of Aisha

**DOI:** 10.3390/brainsci15010094

**Published:** 2025-01-20

**Authors:** Federica Visco-Comandini, Alberto Barbieri, Francesco Mancini, Alessandra Ciolfi

**Affiliations:** 1 Associazione di Psicologia Cognitiva APC e Scuola di Psicoterapia Cognitiva SPC, 00185 Rome, Italy; f.mancini@unimarconi.it (F.M.); alessandraciolfi13@gmail.com (A.C.); 2 Psyché Clinical Center for Transcultural Mental Health, 00100 Rome, Italy; alberto.barbieri@mediciperidirittiumani.org; 3 Department of Human Sciences, Guglielmo Marconi University, 00193 Rome, Italy

**Keywords:** C-PTSD, sensorimotor psychotherapy, cognitive behavioral therapy, trauma, transcultural setting

## Abstract

This case study examined the process of integration of cognitive behavioral therapy (CBT) and sensorimotor psychotherapy (SP) in three-phase trauma treatment with a 32-year-old female Bengali refugee with Complex Post Traumatic Disorder (C-PTSD). The treatment was provided in a Western country. The client’s improvement was assessed by using self-report assessments of post-traumatic, dissociative, and depressive symptoms. Psychodiagnostics’ scores gathered after 2 years of treatment showed significant improvements in post-traumatic, dissociative, and depressive symptoms. Although firm conclusions cannot be drawn due to the limitations of this study, results suggest that integrating CBT and SP in a phase-trauma approach may be an effective treatment for C-PTSD in a transcultural setting. However, replicating and standardizing such preliminary results on larger samples is necessary. **Background/Objectives**: SP is an innovative psychotherapeutic intervention aimed at treating trauma through a bottom-up approach, however, little research exists regarding the efficacy of this psychotherapy. This case study aims to investigate the efficacy of the combination of CBT and SP in treating a C-PTSD patient in a transcultural setting. **Methods**: Three questionnaires were administered to investigate post-traumatic, depressive, and dissociative symptoms. **Results**: Clinical and psychodiagnostic outcomes highlight significant psychological improvements in the patient. **Conclusions:** Although any firm conclusion cannot be drawn because of various intrinsic limitations (i.e., single case study) that straiten our ability to extend these results, this case study suggests that the integration of CBT and SP may be an effective treatment for C-PTSD in a transcultural setting.

## 1. Introduction

In the last years, many asylum seekers and refugees coming from Sub-Saharan countries arrived in Europe. According to the United Nations High Commissioner for Refugees [1], more than 110 million people worldwide have been forced to flee for several causes. Among these, 36 million are formally registered refugees [1]. A recent report released by Medici per i Diritti Umani [2] states that between 2021–2023, over 300,000 migrants and refugees have landed in Italy, embarking from Libya and crossing the Mediterranean Sea. Collected testimonies reported that most of them have suffered severe traumatic experiences such as detention, violence, and abuse in their countries of origin or along the migratory route [2]. Libya represented one of the most cited countries where these violations occurred [2].

Such data are in line with several studies that highlight the traumatic consequences faced by forced migrants, including the heightened risk of human rights violations in their country of origin or along the migration route [3,4]. These violations include instances of conflict involving oppression, persecution, abuse, sexual violence, maltreatment, torture, and homicide [5]. Refugees are exposed to both complex traumas in their countries of origin or along migratory routes and to several post-migratory stressors in the hosting countries [6]. Several studies have shown that refugees face significant mental health challenges [7]. Specifically, post-traumatic stress disorder (PTSD), depression, and anxiety disorders are more frequent among refugees than in the general population [7,8]. PTSD is one of the most frequent mental disorders in such groups [7,9,10]. Due to methodological differences (i.e., sample size, timing of assessment, use of different self-report questionnaires, and different PTSD criteria), the prevalence rates vary substantially between studies. However, post-traumatic symptoms are described among refugees between a range of 9–30% [7,10].

In the transcultural setting, a recently recognized psychological construct is complex PTSD (C-PTSD). The 11th revision of the International Statistical Classification of Disease and Related Health Problems (ICD-11), in 2018, has introduced the diagnosis of C-PTSD whereas in addition to the standard PTSD criteria (re-experiencing, avoidance, perceptions of heightened threat), problems defined as “disturbances in self-organization” are added [11]. C-PTSD is a consequence of complex trauma, which is commonly experienced by refugees [12,13,14]. In addition, refugees are displaced to post-migration environments where it is often difficult to access important sources of social and emotional support for managing distress (e.g., family and friendship networks, work, and leisure activities). Lack of social and emotional support may significantly impact affect regulation, interpersonal relations, and self-concept, which are the core characteristics of C-PTSD. The methodological problems previously described in PTSD studies also have an impact on estimating the prevalence of C-PTSD among refugees. Indeed, Mellor and colleagues [15] recently indicated a considerable variability in the preference rate of C-PTSD among this population, with a rate between 3.0% and 85.5%. It is relevant to be aware of this data to experts in this field to develop systematic psychodiagnostics tools and specific clinical interventions [16].

Psychotherapy has been demonstrated to be an effective tool for treating both young and adult refugees [17]. Recently, research on mental health in refugees has expanded the clinical analysis for a multidisciplinary and integrative approach to therapeutic treatment, including bodywork, spiritual and meditative techniques, and narrative tools of trauma to reconnect the body [18].

Today, the most used treatment for PTSD is trauma-focused psychotherapy, which is a comprehensive term that includes several treatments. Among these, prolonged exposure, eye-movement desensitization, reprocessing (EMDR), and cognitive processing therapy are the most used. Trauma-focused therapies involve both cognitive behavioral strategies and emotional processing. The restructuring of negative beliefs related to the traumatic experience, oneself, and the environment is also included. This modular approach represented the most recommended treatment for post-traumatic symptomatology among refugees [19].

This group of interventions is thought to reduce post-traumatic symptoms such as intrusive memories, avoidant behaviors, alteration of arousal, processing of traumatic memories, and altering maladaptive appraisals of threats [20,21,22,23,24,25]. However, multimodal intervention is the approach that has recently prevailed in the mental health treatment of refugees. Indeed, it has been claimed that focusing solely on trauma might not be fully inclusive to address post-traumatic symptoms, especially when there is a C-PTSD diagnosis. Studies have even argued that implementing exposure methods (such as revisiting traumatic memories) while refugees are in a state of heightened distress could lead to negative emotional responses (for discussion, see [26,27,28,29]).

Another possible approach, which we argue here has the potential to address all aspects of C-PTSD symptomatology, suggests that the treatment of traumatized individuals should help them regain a feeling of security in their bodies [30]. Indeed, an experience becomes traumatic when an individual is unable to escape, defend themselves, or flee, and whatever implemented action does not guarantee a sense of security [31]. In this perspective, body psychotherapies assume that traumatic experiences are embodied in present physical states and automatic behavioral patterns. In this sense, trauma manifests through breathing, body language, sensory experiences, movement, emotions, and eventually cognition [29,32]. Within this framework, the rational (cognitive) brain seems not to adequately manage the sensations, control emotional arousal, and change fixed action patterns, which frequently represent problems experienced by traumatized individuals [30]. Body experience related to trauma has not been widely investigated [32,33,34,35,36]. Only recently, the need to demonstrate effectiveness in approaches where the body represents the main therapeutic tool to address trauma and concomitant emerged. Research on yoga [36], somatic experiencing [37,38], and sensorimotor psychotherapy [39,40] has been recently developed.

Sensorimotor Psychotherapy (SP) is a somatic-based psychotherapy specifically developed for trauma and is designed to deal with problems stored in somatic memory [31,41]. This approach is developed from clinical practice and inspired by attachment theory, trauma, and neuroscience [42]. It is an integrative treatment that uses physical, emotional, and cognitive processing of the treatment of trauma, focusing mainly on somatic process [32,43], and it is based on the understanding that the body remains affected by implicit memory fragments linked to traumatic events that indicate threat even without immediate danger [43].

SP aims to integrate sensorimotor processing with cognitive and emotional processing, centering on the body as the key focus of the treatment [32,43]. This intervention is possible thanks to the ‘bottom-up’ approach, which allows for the treatment of the brain’s automatic and involuntary functions that underlie post-traumatic responses [32]. This approach is significantly different from ’top-down’ traditional therapies that mainly focus on reducing reactions to trauma since it uses the body as the main channel to elaborate trauma [44]. This psychotherapy is used to treat simple PTSD, C-PTSD, disorganized and insecure attachment patterns, and severe dissociative disorders [42,45].

Within this therapy, it is possible to identify three major steps: (i) increase the ability to focus on movements, impulses, and physical sensations in a safe setting (therapeutic context); (ii) develop somatic resources to process traumatic memory and re-experience the trauma in a new framework [32]. In the third phase (iii), skills and abilities are combined to attain success in daily life, fostering a renewed sense of self that is resilient and adaptable [32].

The integration of top-down therapies and bottom-up therapies, such as this approach, may represent a valid tool to treat post-traumatic symptoms, especially in a non-western context where narrative may represent an obstacle in the process of trauma elaboration. Indeed, recent systematic reviews have highlighted the urge to rethink a transcultural and holistic approach [46] and psychosocial perspective [47,48,49] to treat traumatized refugees.

As Pat Ogden recently stated, commonly recognized mental health frameworks that form the basis of current evidence-based psychotherapy methods are primarily rooted in Western perspectives on illness and health, often undervaluing practices from other cultural traditions [50]. Therapists should recognize the importance of critically examining culturally bound assumptions while engaging in open dialogue with alternative perspectives on symptoms and pathways to well-being [50]. The integration and adaptation of culturally sensitive approaches to understanding illness presentations and therapeutic interventions remain insufficiently explored at both theoretical and practical levels. This limitation can reduce the effectiveness of treatment for individuals who do not align with mainstream norms [50]. In this sense, using somatic therapies may reduce bias and meanings linked to the Western perspective on mental health and reduce the cultural gap among cultures.

Employing somatic tools in trauma treatment provides a holistic approach to addressing trauma by integrating body-centered methods in the healing process. Particularly, implementing such integration within a transcultural context offers an innovative approach and a valuable opportunity to address deeply rooted and often inaccessible traumatic experiences. Somatizations among refugees are a complex phenomenon as they encompass various aspects tied to personality, psychopathology, and migratory processes. The bodily dimension of symptoms in this population often conceals pre-existing vulnerabilities, traumatic experiences tied to the journey, different cultural codes for interpreting experiences, and collective memories. This intricate interaction of factors makes it essential to approach this phenomenon with priority and sensitivity [51]. For these reasons, integrating the bodily dimension into the treatment of complex post-traumatic symptoms may represent a useful and effective tool for treating such psychopathologies among refugees. Indeed, the integration could represent a response to the challenges related to cultural biases.

In this sense, developing a new integrated clinical intervention aiming at modifying both the dysfunctional cognitive strategies and the somatic activation linked to trauma may represent an innovative clinical approach.

This study aims to enrich the present body of knowledge by exploring the combined use of Cognitive Behavioral Therapy (CBT), trauma focus therapy, and PS in a cross-cultural setting consisting of a Bengalese refugee client, a cultural mediator, and an Italian therapist.

We opted for CBT as an evidence-based approach to address dysfunctional beliefs, foster functional behavioral strategies, facilitate extinction learning, reducing avoidance behavior and intrusive symptoms alongside SP, a body-centered therapy aimed at processing trauma, particularly focusing on the somatic expression of trauma.

To the best of our knowledge, no studies to date have studied the cross-cultural impact of PS in severely traumatized clients.

In this clinical case, we outline the clinical treatment of a 32-year-old female Bengalese-speaking refugee with C-PTSD. In the first section, we will describe the three-phase treatment process, and afterward, we will present scores of psychodiagnostics questionnaires at the beginning and after 2-years treatment through the analysis of the PTSD Checklist for DSM-5 (PCL-5 [52]), Hamilton Depression Scale (HAM-D [53]), Dissociative Experience Scale-II (DES-II [54]).

## 2. Materials and Methods

### 2.1. Procedure

The client took part in the case study after providing written informed consent. She understood and consented to the use of the therapists’ notes, psychotherapy process, and assessments for the single case study. Separate consent was acquired for the publication of the drawing.

We used a fictitious name for the client (i.e., Aisha) and omitted all information that was not strictly necessary for understanding the case. The treatment process was documented based on the therapists’ case notes and discussions between the therapist and supervisor.

### 2.2. Client

Aisha is a 32-year-old woman who is married and has two daughters. At the beginning of the therapy, both of her daughters were living in Bangladesh. At the time of her initial clinical interview, she was residing in a reception center in Italy. Aisha said that she had to flee her country to “be safe” from the abusive relationship with her ex-husband. She grew up in a rural area of Bangladesh in a low socio-economic context. She referred to being physically abused by her father when she was young and forced to marry a 28-year-old man when she was 13. Since the forced marriage, she was psychologically, physically, and sexually abused by her husband. During this period, two daughters were born (respectively 14 and 12 years old at the time of the first consultation). After about 9 years of violence, Aisha was able to receive support from her mother, thus escaping the abusive and violent familiar context. Therefore, she moved into the family house located in another village. However, after 1 year, she realized she could not offer her daughters the economic support she was thinking of. She then left Bangladesh looking for a better economic condition and arrived in Italy. However, once in Italy, she was sexually exploited by the smuggler who helped her to arrive in this country. In 2021, she managed to escape from the exploited network and, since then, found shelter in a protective reception center.

### 2.3. Setting and Psychotherapists

The case was conducted at the Psyché Clinical Center for Transcultural Mental Health, operated by the medical humanitarian NGO Medici per i Diritti Umani (MEDU) in Rome (Italy). Dr. F.V.C., under the supervision of A.C., both certified cognitive-behavioral therapists (F.V.C. and A.C.) and certified sensorimotor psychotherapist (A.C.), conducted the psychotherapy -weekly, 60-min sessions. The clinical sessions were held from July 2022 until the present moment (June 2024). The first 12-month sessions (July 2022–July 2023) were held in Bengali, and the translation was done by an interpreter-certified cultural mediator native of Bangladesh. The sessions were conducted in Italian in the following stage (September 2023–present). This shift was made at the client’s request to interact exclusively with the therapist, as her language proficiency made this feasible. Furthermore, the extensive use of SP techniques reduced dependence on language, using it primarily as an integrative tool in the therapeutic process and significantly reducing the risk of misunderstandings. To support this, the therapist also introduced a somatic vocabulary to the client, enhancing her ability to understand and interpret physical sensations.

## 3. Results

### 3.1. Presenting Complains

While completing the necessary documents to initiate Aisha’s training in sewing, social operators of the reception center where she was living noticed that she struggled to concentrate during Italian classes, became tearful, missed appointments, and often suffered from physical symptoms (mainly headaches). As a result, reception center operators referred Aisha to MEDU Psychè Center (Rome, Italy) to treat such symptoms. Aisha arrived promptly for her intake assessment at Psychè Center. As she could not speak Italian, a Bengalese cultural mediator was provided. Aisha presented herself as an appropriately dressed and groomed 32-years-old Bengalese woman. She referred to anxious states, difficulty breathing, and severe insomnia. In addition, she manifested intense fatigue and a sense of loss of energy, diminished ability to concentrate, and physical pain. In particular, she manifested persistent migraine (“I can feel the veins in the brain”). She reported numerous complaints of physical and emotional distress, particularly dissociative states (“at night, when I lay down on the bed, I stop feeling my legs, I can’t move them, it lasts several minutes”) and depressive states. A sense of guilt emerged regarding her family still living in Bangladesh and self-critic for the decision to flee from her country (“I shouldn’t have left Bangladesh If I had accepted everything he [husband] wanted, I wouldn’t be in this condition”).

Throughout her intake interview, Aisha struggled to tell her story in detail. She referred to being abused during her childhood by her father and beaten and sexually abused by her husband.

Despite her discomfort during her intake, Aisha expressed a strong desire to heal; she stated that she wanted to “feel normal”.

Aisha denied experiencing aggressive impulses and declared no alcohol or substance abuse.

### 3.2. History

Aisha grew up in a low-income family living in the countryside in northern Bangladesh. She was living with her parents and three older brothers. She says she was beaten by her father when she was 5 years old. At the age of 13, she was forced to marry a 24-year-old Bengalese man. During her marriage, she was psychologically, physically, and sexually abused by her husband. Over the years, she gave birth to two daughters (at the time of the assessment, 11 and 13 years old, respectively, living in Bangladesh with their grandmother). When her father died, she escaped from her husband and returned to her family with her daughters. In search of a better life, she started looking for a job outside the country. She was contacted by a Bengali man offering a job as a cook in Italy. She trusted him, accepted the proposal, and flew to Italy in 2021. However, once in Italy, she was forced to enter into a prostitution network.

### 3.3. Case Conceptualization

Aisha’s psychological profile has been conceptualized within the theoretical framework of the “structural dissociation of the personality” presented by Van der Hart [55]. Specifically, the authors suggested that trauma entails a certain level of dissociative fragmentation of personality that can be divided into (i) innate action systems of daily life and (ii) systems of animal defense responses. The authors defined the part(s) of the personality guided by the so-called Apparently Normal Part of the Personality (ANP) and the parts guided by the traumatic responses as the Emotional Parts of the Personality (EP). Specifically, the single EP can be differentiated into sub-parts, defined as the Fight, Flight, Freeze, Submit, or Attach for Survival parts. Each EP is detached, and it is connected to a distinct sense of identity [56]. The ANP refers to the rationale, present-oriented, and grounded parts of the individual, while the EP is triggered when strong emotions take over during the reliving of trauma [57]. The EP often experiences traumatic memories as overwhelming, involving images, feelings, and physical actions [56]. They might also encompass misunderstandings from the moment of the traumatic event. Indeed, traumatic memories may often be corrupted [56]. The authors identify three fundamental levels of structural dissociation: primary structural dissociation, secondary structural dissociation, and tertiary structural dissociation. The primary structural dissociation may include trauma-related disorders such as Acute Stress Disorders, simple PTSD, simple dissociative amnesia, and simple somatoform dissociative disorders [57]. Within this level, a single dissociative part of the personality (i.e., EP) exists, which avoids trauma-related cues, and a part mediated by defense (i.e., ANP). The secondary structural dissociation is characterized by several emotional parts (i.e., EPs), which are linked to trauma, and one ANP. This level might include severe complex and chronic trauma-related disorders, such as C-PTSD/DESNOS [58]. The tertiary structural dissociation is characterized by the presence of several dissociative parts (i.e., EPs) and several parts of the apparent normal functioning (i.e., ANPs). It represents the more severe form of dissociation that may be manifest among DID and more severe trauma-related disorders [57].

The core impediments to resolution for such dissociated parts emerge in relation to trauma-related triggers experienced in daily life. Indeed, clients with such a level of dissociation manifest difficulty regulating impulsivity, and intimacy and closeness are characterized by fear and mistrust in relationships. Within this conceptualization, ANP systematically avoids thinking about traumatic memories, while the trauma-associated parts remain persistently focused on threats, grief, fear, and inability to regulate dysregulated autonomic activation. This internal fragmentation interferes with the integration between the past and present.

**Figure 1:**
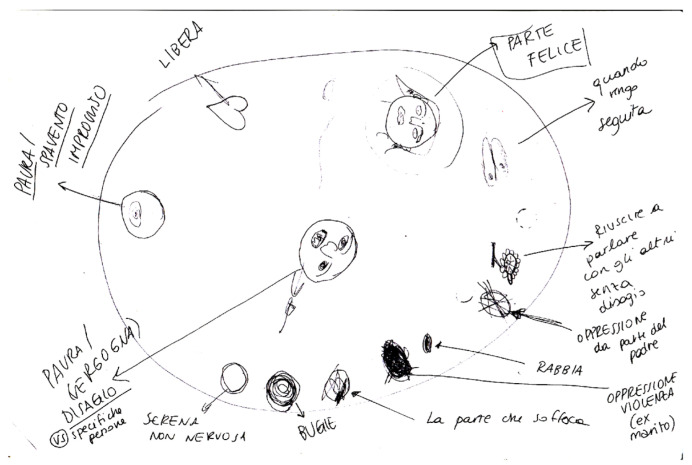
Drawing of the parts observed by Aisha. Translation of Italian words: Libera = be free; parte felice = happy part; quando vengo seguita = when I am followed; Riuscire a parlare con gli altri senza disagio = be able to speak with others with confidence; Oppressione = Oppression; Rabbia = anger; la parte che soffoca = the part (of my self) that suffocate; bugie = liers; Serena ma non nervosa = serene not nervous; Paura/Vergogna = fear/Shame; Paura/Spavento improvviso = Fear/Shock).

We conceptualized Aisha’s profile within the Secondary Structural Dissociation Model. We asked her to describe her internal functioning following the personality divisions suggested by van der Hart [57]. The description that emerged shows a significant fragmentation of personality. In particular, the ANP has been characterized by stability, safety, and engagement in the therapy. The EP has been highlighted and described based on the symptoms/activations that Aisha outlined (Figure 1).

**Figure 2:**
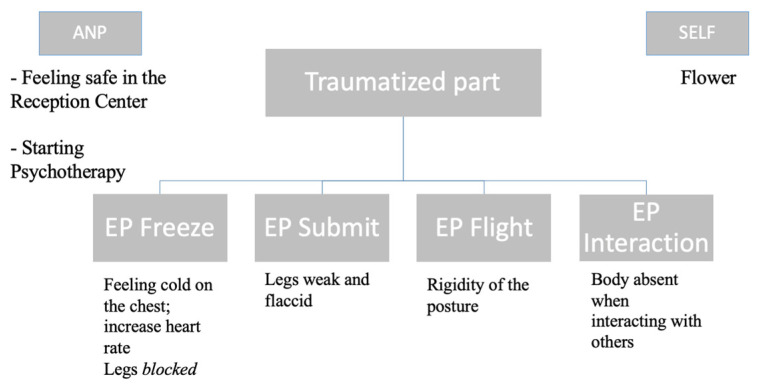
Conceptualization of emotional parts of Aisha according to the Structural Dissociation. Model ([55] Van Der Hart, 2004).

We identified four emotional parts (EP) and the corresponding somatic indicators: (1) the frozen part was characterized by a cold feeling in the chest, an increase in heartbeat when she was lying down in the bed, and her legs were blocked; (2) The submit part was characterized by weakness and flaccidity of the legs; (3) the rigidity of the posture characterized flight part; (4) the attach for Survival part (relational level) was characterized by rigidity of the chest and freezing responses when speaking with others (Figure 2).

### 3.4. Phase of the Therapy

An ongoing discussion persists about whether individuals with C-PTSD should undergo phase-based or single-phase psychological therapies [59], as they often encounter obstacles in obtaining and completing effective and timely treatment [59,60,61].

**Figure 3:**
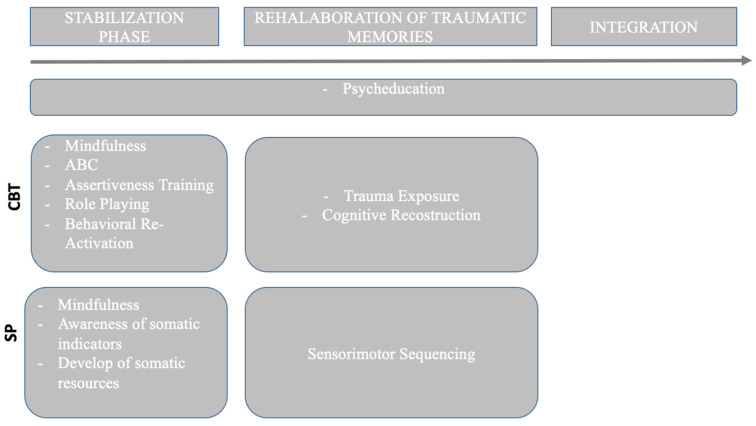
Timeline of the Therapeutic intervention.

A recent review highlights several studies that identified the phase-based approach as the best intervention for C-PTSD [59], with particular attention to the stabilization phase [62]; in addition, multi-approach treatment is suggested to encounter all the pervasive characteristics of this disorder. Typically, Phase 1 focuses on stabilization, building, and strengthening self-regulation skills, such as ensuring the client’s safety, enhancing emotional expression, fostering positive self-perceptions, addressing guilt and shame, and improving interpersonal relationships [63]. In Phase 2, the traumatic memories are revisited with the goal of reliving the traumatic events within a secure setting. Phase 3 focuses on strengthening the client’s emotional, social, and interpersonal skills [63,64]. It is important to highlight that the client can move back to previous stages, depending on the development of the skills, rather than time [65]. We have adapted the phase-based intervention to integrate CBT and SP techniques to maximize the efficacy of the treatment. In Figure 3 a schematic description of the phases of the intervention.

#### 3.4.1. First Phase of the Therapy

The stabilization phase represented the first phase of the intervention. In this phase, the therapist mainly focused on improving the client’s affect regulation, somatic awareness, and development of somatic resources, forming a therapeutic alliance, as well as creating the Psyché Center as a safe place for the client.

During the first sessions, the therapist educated Aisha about trauma and dissociation through psychoeducation intervention. Psychoeducation has represented a fil rouge during the whole therapy since, with traumatized clients, it is recommended to bring them back to internal functioning continuously and to explain the different activation of the emotional parts. In this phase, the therapist tried to stay focused on helping Aisha notice the pattern: the relationship between triggers and symptoms, dysregulation, and the emergence of different EPs. During the assessment phase, Aisha drew a picture of her internal functioning (Figure 1).

Aisha identified several parts of her internal functioning and, together with the therapist, classified them based on the Structural Dissociation Model [55], as explained before (see also Figure 2). The problems reported by Aisha can be summarized into three main clusters: (i) depressive states and negative beliefs about herself and the world, (ii) pervasive post-traumatic symptoms, and (iii) dissociative states. Psychodiagnostics questionnaires were administered during the first phase and re-administered at the end of the intervention (see Table 1 for the results of the psychodiagnostics results). In detail, the PCL-5 to investigate post-traumatic symptoms, the DES-II to investigate dissociative symptoms, and the HAM-D for depressive symptoms. These questionnaires are not available in Bengali. Therefore, we asked a cultural mediator who has received specific training in psychological intervention for post-traumatic symptoms to translate them for the client.

Psychodiagnostics results highlight severe post-traumatic symptomatology (PCL-5, Score = 61), moderate depressive symptoms (HAM-D, Score = 21), and dissociative symptoms associated with probable PTSD (DES-II, score = 28.5). Depersonalization/derealization symptoms and absorptions were of significant interest, which was represented, among the dissociative states, as the most invalidating. A suspected diagnosis of C-PTSD was determined based on the ICD-11 International Trauma Questionnaire (ITQ [66]) criteria, a self-report tool specifically developed to assess both PTSD and CPTSD symptoms. A diagnosis of C-PTSD necessitates PTSD and specific thresholds across all three DSO clusters. We used the same PCL-5 items to investigate DSO symptoms as previously published [14]. In detail, we used 7 items derived from the PCL-5: (i) Affective Dysregulation (AD): PCL-5 (item [11,14,15,16]); PCL-5 (item [14]); (ii) Negative Self-Concept (NSC): PCL-5 (item [9,10]); (iii) Disturbed Relationships (DR): PCL-5 (item [13]). Aisha reported, in addition to meeting the criteria for ICD-11 PTSD, the following scores for DSO symptoms: AD = 7 for hyperactivation, AD = 3 for deactivation, NSC = 7; DR = 3. This procedure has been previously used [14].

The client shared several highly traumatic experiences, which can be identified as complex trauma: domestic violence, physical, psychological, and sexual abuse during her marriage, and sexual exploitation upon her arrival in Italy. The combination of the psychodiagnostics outcomes and the history of complex trauma support the possible diagnosis of C-PTSD.

Through cognitive-behavioral intervention, the therapist strengthens ANP. This intervention was aimed at reducing the sense of uselessness and incapability that Aisha experienced and maintained depressive symptoms and pathological beliefs. Specifically, Aisha was taught to identify the cognitive foundations of her maladaptive behaviors and distressing emotions by practicing various exercises using the ABC technique [67], which investigates the antecedents, cognition, emotions, and behaviors of an event in the client’s life. In particular, the goal was to highlight the core pathological beliefs that activate specific emotional patterns. In addition, Aisha was trained to observe somatic symptoms linked to the traumatic memories, and through cognitive techniques, she was able to reduce the effects of such activation, for example, through modifying catastrophic cognitions about somatic sensations.

In addition, a behavioral activation (BA) has been performed to reduce negative self-evaluation and to increase self-efficacy. BA is a research-supported, time-constrained, and systematic psychotherapeutic approach designed to enhance behaviors that connect the client with environmental rewards and reduce behaviors that prevent access to positive reinforcement, such as inactivity and avoidance [68]. To increase assertiveness, Assertive Training [69] was performed together with role-playing linked to working interactions.

Mindfulness-based exercises, taken from the Mindfulness Based Stress Reduction (MBST, [70]), have been performed to increase the somatic awareness and the ability to remain present, along with grounding techniques to assist her in remaining focused in the present moment. Aisha was instructed to practice mindfulness with an emphasis on focusing inward to observe what is happening in her mind and her body. Exercises focused on breath, body scans, and walking meditation were performed.

At the sensorimotor level, the therapist taught Aisha how to differentiate the ANP from the traumatized and not integrated part, guiding the client in mindfulness-based self-awareness methods to distinguish the immediate bodily sensations arising from various traumatized parts, ultimately learning to recognize them. In this phase, the therapist tried to integrate the two approaches (CBT and SP), highlighting the relation between the identified emotional parts and cognitive beliefs. Specifically, each EP, previously somatically identified, was associated with the cognitive beliefs underneath (1) the frozen part with vulnerability and insecurity; (2) The submitting part with feeling powerless; (3) the flight part with the constant feeling in danger; (4) the attach for Survival part (relational level) with mistrust of others (see Table 2).

This integration was made to strengthen the awareness of her internal functioning.

Subsequently, the therapist and the client worked together to develop somatic resources for stabilization, focusing on developing self-regulatory skills that keep arousal levels within the optimal range of tolerance [32]. During this phase, through psychoeducation, identifying her triggers, and mindful awareness of associated arousal and defensive systems, Aisha learned to recognize when her arousal surpassed the window of tolerance and apply resources that helped her achieve greater stability [32]. Somatic resources are capabilities that arise from physical experience but impact psychological well-being. These include physical functions and abilities that promote self-regulation and foster both physical and mental health and competence [32]. These resources are implemented to increase her window of tolerance. Aisha developed several somatic resources to self-regulate her hypo and hyper-arousal for each EP. In particular, for the frozen part, the therapist and client were seated, legs crossed on the ground, and Aisha implemented a self-touched action in order to reduce the feeling of freeze experienced on the legs in order to re-activate the flaccidity and weakness of the legs characterizing the submit part, the therapist supported the client to walk through the therapy room, helping Aisha feeling the strength of the legs and the muscles; the somatic resources used to reduce the rigidity of the posture was gently swaying from side to side; grounding exercises and self-touched such as the touch of the face were implemented to reduce the dissociative states such as not feeling present in the interaction with others.

In this phase, pharmacotherapy has been prescribed to reduce insomnia.

#### 3.4.2. Second Phase of the Therapy

Once Aisha had reached stabilization in terms of arousal and self-regulatory skills, the therapist proceeded together with the client to explore the traumatic memories in order to favor the rehabilitation of stressful experiences. To implement the elaboration of the traumatic memories and the reconstruction of pathological beliefs, both narrative exposure to traumatic memories and SP interventions were implemented.

Through the float-back technique, Aisha was able to recollect specific intrusive and traumatic memories regarding experiencing the violence suffered by her husband. The essential feature of this technique is helping the client to connect present problems with past events using specific questions aimed at focusing on the mental and emotional states of the upsetting situation and helping him/her make an association with previous traumatic experiences linked to those feelings. The exposure technique was used to re-elaborate these episodes in which Aisha experienced profound suffering. In addition, a cognitive reconstruction intervention has been implemented to modify pathological beliefs connected to these traumatic experiences.

Then, because most of the traumatic memories were not entirely remembered by Aisha as a unified, coherent personal story, Aisha was unable to deal with the clinical pathological manifestation of such memories, making them even more unintegrated [32]. The manifestation of such memories emerged through a series of repeated and rigid activation patterns, “idéeas fixes” (Janet cited in [71]). Indeed, even though narrative memory is partially missing, sensations, intense emotions, and maladaptive physical actions are still available [31]. This disintegration between the rational and somatic parts of the self makes it hard for Aisha to understand the link between her bodily sensations/activation (somatic memory) and the conscious narrative elements accessible to her [35,36,56]. Within complex trauma theorization, Fisher [72] describes this kind of fragmentation as “compartmentalization under stress” (p. 22).

Thus, during this stage of treatment, the therapist employed sensorimotor sequencing to address the overwhelming and unsettling aspects of bodily experience [32]. Sensorimotor sequencing is a therapeutic technique to treat bodily activation related to trauma where the therapist supports the client in mindfully tracking the bodily sensations of such activation until they settle [73]. This process is mediated by dual awareness [32], a specific state processing where the client simultaneously looks into the somatic states related to the traumatic memory whilst mindfully observing the unfolding experience [73].

In this context, the therapist pushed Aisha to re-activate feelings and sensations of her primary symptom, which was an intense headache. By mindfully noticing the physical manifestation of the nonverbal memory, in this case, it was a mindful description of the feeling she was experiencing (itching and burning feelings), the body sequencing intervention began with “mindfulness of present moment organization of experience” [73] whereby awareness of sensation through the description of somatic characteristics is observed. This engagement helps identify conditioned behaviors known as action tendencies, which can be resolved through the sequencing process [73]. Aisha learned to tolerate somatic activation, tracking physical sensations as they move through the body. Indeed, Aisha was able to unfold and express the frozen, unexecuted movement pattern of flight, which was not allowed to manifest at the time of the actual trauma. Moreover, through the therapist’s assistance, Aisha was able to follow the sequencing of the feeling that moves through the body, starting from the head and passing through the face, neck, arms, and hands. The sequencing reached a specific set in which Aisha felt her hands huge and heavy (“I can’t move them; they are blocked”). Slowly, Aisha was able to open her hands and start moving them in the air until a feeling of lightness and power was established.

Aisha experiences satisfaction and pleasure after completing such actions; these feelings were visible through newfound self-assurance, boosted energy, and changes in posture. Beyond these physical changes, a sense of safety and self-efficacy in her ability to act and deal with the difficulties in the present emerged. Once the sensorimotor activation is entirely processed, consequentially, emotional and cognitive components associated with that memory tend to transform [73]. This shift emphasizes the value of working bottom up. The new awareness of the body, finally free from disturbing traumatic memories, can now lead to the modification of cognitive beliefs. Indeed, Aisha started to feel stronger, powerful, and safe, and she started (among the therapeutic relationships) to establish intimacy at the interpersonal level.

#### 3.4.3. Third Phase of the Therapy

This phase of the therapy was aimed at reinforcing therapeutic progress and supporting Aisha in gaining more independence in her daily. During this phase, Aisha progressively increased her social and professional connections; she was enrolled as a cleaning lady in an elementary school with a permanent position. In addition, with the support of another NGO, Aisha allowed her daughters to join her in Italy. Therefore, in May 2023, the two daughters arrived in Italy and moved with Aisha to another reception center for victims of domestic violence. Both of her daughters were supported and helped in starting middle school. Over 6 months, the number of sessions was reduced to one every two weeks.

### 3.5. Qualitative Assessment of Psychotherapy Outcome

In June 2024, psychodiagnostics questionnaires were re-administered, and all the indicators related to post-traumatic, depressive, and dissociative symptoms were reduced and were below the cut-off. In detail, PTSD symptoms decreased to 37 points on the PCL-5, falling below the clinical threshold (>33, Aisha obtained 24), no longer fulfilling the criteria for C-PTSD. Aisha’s depressive symptoms severity was reduced by 11 points, shifting her score to the mild range of depression. Dissociative symptoms were significantly reduced, moving from 28.5 to 13.3. However, affective dysregulation, especially in terms of hypo-activation, was still present. Living together with her daughters, on the one side, gave Aisha emotional benefit; on the other side, it represented a trigger for her pathogenic beliefs regarding her sense of incapability.

## 4. Discussion

This single case study is the first to describe a combination of CBT and SP treatment for a C-PTSD client in a transcultural context. CBT and SP intervention were used alternately during the first phase (stabilization phase) and integration of traumatic memories phase.

The case report aimed to provide preliminary data on the efficacy of the integration of these two psychotherapies. In particular, the intervention has been divided into phases where the client progressively learned how to deal with post-traumatic, depressive, and dissociative symptoms. The internal functioning of the client has been conceptualized within the framework of the Secondary Structural Dissociation Model [57]. We have identified a single ANP and four EPs representing the dysregulated and non-integrated emotional parts that emerged as a consequence of complex traumas. The identified EPs were freeze, submit, flight, and attach for survival parts. For each part, specific somatic indicators (somatic level) and the corresponding pathological beliefs (cognitive level) were specified and shared with the client. Since the client showed pervasive post-traumatic symptomatology, a phase-based approach has been provided, integrating both cognitive behavioral techniques and a sensory-motor approach.

The first phase of the therapy was aimed at providing stabilization. Both CBT and SP techniques were used together with Psychoeducation and Mindfulness exercises. In particular, mindfulness techniques were implemented to progressively recognize the moment-by-moment body inputs coming from different traumatized parts, ultimately learning to recognize them, and somatic resources were developed to increase the window of tolerance. Mindfulness exercises were provided during the initial phase of the therapy to help Aisha’s ability to be present. Psychoeducation was provided since the beginning of the therapy and during all the sessions, implementing Aisha’s ability to identify each EP through the activation of specific somatic indicators and highlighting the underlying cognitive beliefs. This phase represented one of the integration of SP and CBT.

Mindfulness exercises were used since several studies highlighted clinically significant therapeutic effects among refugees suffering post-traumatic symptoms [9,74]. In particular, the main effect has been observed in all PTSD clusters, with particular attention to PTSD hyperarousal [75]. This data is particularly interesting since, in our client, we observed a significant reduction mainly in the alteration of the arousal cluster since the score of the alteration of the arousal cluster decreased from 15 to 7.

Regarding CBT techniques, ABC [67] and Assertiveness Training [68] were provided to increase cognitive awareness and assertive behaviors and strengthen the ability to express her needs and intentions. Cognitive reconstruction has also been performed to modify maladaptive beliefs. CBT was used since these are relatively well-researched and support interventions for traumatized refugees [76,77]. Indeed, the treatment centers on modifying dysfunctional beliefs in trauma survivors to alleviate psychological distress and enhance functioning [78]. CBT aimed at reducing post-traumatic symptoms by facilitating extinction learning and adjusting distorted interpretations of risk and negative beliefs [19,20,21,22]. Indeed, several studies show a significant effect of CBT on post-traumatic symptoms among refugees [79].

During the second phase of the therapy, narrative exposure of traumatic memories and sensorimotor sequencing were provided to favor the rehabilitation process of traumatic events.

Several studies demonstrated how Narrative Exposure Therapy (NET) to traumatic events represents a valuable tool for treating post-traumatic symptoms [80], especially among survivors of violence [81,82,83]. Indeed, exposure to traumatic memories enhances activity linked to cortical top-down control of focus on negative stimuli, thus reducing PTSD symptoms [84]. In this case report, the entire procedure of NET was not provided to the client since several traumatic memories were not accessible. However, after the stabilization phase, Aisha was able to access specific traumatic memories related to experiences of violence and abuse, and for those, narrative exposure was provided. The trauma exposure to specific memories helped Aisha to tolerate the physiological activation related to those memories, helping her in the process of rehabilitation.

Sensorimotor sequencing represents one of the tools used by SP to favor the elaboration of trauma. Limited research has investigated the effectiveness of SP in addressing traumatic memories, mainly in group interventions [39,40]. Gene-Cos and colleagues [39] implemented SP group intervention addressing different sets of symptoms, including autonomic arousal, implicit memory, strategies for managing arousal, directing focused attention, setting boundaries, and practicing essential skills for chronically ill populations hosted in the Trauma Clinic. Results showed highlighting statistically significant changes in PTSD symptoms, depression, and general social functioning. Langmuir and colleagues explored the efficacy of the SP group on women who experienced childhood abuse, and results showed significant improvement in body awareness, level of dissociation, and receptivity to soothing [40].

However, no specific studies have been carried out to investigate the efficacy of sensorimotor sequencing. Indeed, this case report represents the first attempt to demonstrate the clinical impact on post-traumatic symptoms.

Regarding somatic therapies, few studies have shown efficacy in reducing post-traumatic symptoms by providing individual support in various traumatic contexts, including the 2004 tsunami [85], war [38], tornados [86], earthquakes [87], treating PTSD [88] and painful disorders [38]. However, only two qualitative studies show the effect of somatic experience in adult refugees [89] and woman refugees [90].

The last phase was characterized by the consolidation of both cognitive and somatic tools she has developed. Aisha was supported in the integration and autonomy process. Psychodiagnostics descriptive results show clinical efficacy in post-traumatic, depressive, and dissociative states, supporting the efficacy of this intervention.

The integration of CBT and SP has been made for several reasons, including (i) the severe fragmentation of the internal functioning of the client, which significantly interferes with the possibility of initially working “cognitively” to treat traumatic memories; (ii) negative effects and risk factors linked to the use of CBT alone on PTSD/CPTSD; (iii) and the current indication regarding the three-phase treatment for C-PTSD, where it is stressed to implement a tailored stabilization phase to enhance clients’ sense of safety, engagement, and efficacy.

About the first explanation, the systematic use of SP techniques during the stabilization phase has represented the key to accessing the declarative memories later. Increasing somatic awareness and the self-efficacy to self-regulate her dysregulation of arousal was necessary to allow her to “cognitively” approach the trauma.

Regarding the negative effects of CBT, several studies showed the limits of CBT in the treatment of severe post-traumatic symptoms since CBT necessitates that fundamental cognitive functions are intact to effectively achieve treatment objectives (i.e., modification of pathological beliefs) [91]. In CPTSD, such processes may be corrupted due to pervasive symptomatology (affect dysregulation, settled negative beliefs), as happened in our client, who was manifesting attention deficit and profound negative beliefs. Affect dysregulation, which was particularly evident in our client due to her traumatic experiences during childhood, significantly interferes with the application of exposure or cognitive reconstructing and represents a risk factor for the development of PTSD and maintaining PTSD symptoms [92]. Recently, authors emphasized the emergence of iatrogenic effects when using trauma-focused techniques to treat PTSD [93,94]. Studies suggest that to reduce the negative effects of CBT on PTSD, clinicians should consider the integration of modules focusing on emotional regulation at the initial stages of the treatment. In fact, some clients may need to practice affect regulation before addressing their trauma [95], while others may benefit from skills training (i.e., STAIR, 95) or mindfulness and crisis planning [96].

Regarding the third reason, it has been widely discussed that individuals with CPTSD tend to benefit significantly more from trauma-focused treatments when they first undergo a stabilization phase [97]. Trauma-focused interventions were found to be most effective for alleviating PTSD symptoms, although outcomes differed across various subgroups [98]. A recent review emphasizes the efficacy of multi-component interventions which incorporate more than one therapeutic modality. Studies suggest that such integrated approaches are particularly effective in addressing both PTSD symptoms and disturbances in self-organization [62]. In this sense, the integration of different psychotherapeutic approaches (CBT and SP) can be viewed as a multimodal intervention, which represents the dominant refugee mental health treatment [99]. Indeed, the multimodal approach represents a tool to address the complexity of psychological reactions that may occur following complex trauma, as well as subsequent post-migration stressors such as psychosocial stressors, physical health problems, and resettlement and acculturation challenges [100]. Studies have argued that a singular treatment may not be sufficiently effective in addressing these diverse and complex needs [27,28,29]. Recently, O’Brian and colleagues [16] have observed that there is a significant lack of thorough psychological evaluations and treatments for refugees and asylum seekers, underscoring the necessity for a culturally psychological assessment, diagnosis, treatment strategy, and intervention tailored for those experiencing embodied trauma, following a humanistic, person-centered [101,102], psychosocial [46,47,48] and transcultural perspective [103,104]. The origins of this approach (i.e., multimodal approach) stem from the recognition that refugees are subjected to a broad array of both pre-migratory and post-migratory stressors [105,106]. Accordingly, the psychotherapy intervention to treat mental disorders among refugees requires a variety of clinical tools to address the complexity of post-traumatic symptomatology. In this sense, the urge to develop an efficacy approach emerges.

Several studies show high comorbidity between PTSD and depression in refugees and more severe chronic pain resulting from trauma-related psychological distress [70,96]. Indeed, the psychopathology of the treated Bengalese client is in line with the current data regarding the main disorders related to refugees [107]. A recent review revealed that approximately one-third of refugees experienced clinically significant psychological distress, with about one-fourth meeting the criteria for a psychiatric diagnosis, most commonly PTSD. Furthermore, the review identified that the number of traumatic events experienced was a significant risk factor for both psychological distress and depressive symptoms [108]. Studies have identified pre-migration, migration, and post-migration phase-specific stressors to develop psychopathologies. Particular attention has been given to post-migration factors such as the critical initial settlement in the host country, the integration process, and challenges to immigration status. Indeed, depressive symptoms seem to be mainly related to these factors [71]. Indeed, several studies showed an increased risk for the development of depressive symptoms as time progresses after a traumatic event [105], and other studies found a negative correlation between post-traumatic growth and depression [109,110].

In summary, CBT has been widely applied in treating post-traumatic symptoms, particularly through exposure techniques. Its primary effects include reconstructing dysfunctional beliefs, facilitating extinction learning, overcoming avoidance behavior, and reducing intrusive symptoms [20,21,22,23,24,25]. However, it has demonstrated limitations, including negative effects [101] and iatrogenic factors [93,94]. However, within this approach, the somatic dimension of trauma is not systematically considered. This may lead to the persistence of specific somatic symptoms or dysregulated reactions to triggers, resulting in the client’s suffering. To mitigate this risk, body psychotherapies could help address this gap. Specifically, SP, a newer body-centered psychotherapy for trauma, has shown promise, with limited research indicating increased body awareness and a reduction in post-traumatic and dissociative symptoms [39,40]. At the clinical level, the primary effects of this approach relate to body awareness and the development of somatic resources that support the client in self-regulation. However, the limited research on this approach (i.e., no clinical trials investigate the effects of SP) prevents the individual use of this approach.

Aisha presented with severe somatoform symptoms along with post-traumatic symptoms primarily related to arousal dysregulation; therefore, we believed that integrating “standard” trauma therapy together with sensorimotor psychotherapy could address these diverse and pervasive symptoms. For these reasons, we opted for the integration of these two psychotherapies. The use of multiple approaches (in this context, CBT and SP) for treating C-PTSD aligns with the current trend of employing multimodal strategies for post-traumatic psychopathologies [62], especially when working with refugees [99].

To our knowledge, there has not been any clinical case report or systematic review that has investigated the impact of the integration of multiple psychotherapeutic approaches, such as CBT and SP, in a non-western patient. The therapy was applied in a Western country, and the setting consisted of a Bengali refugee patient, a Bengali interpreter/cultural mediator, and one Italian therapist. Indeed, one of the most compelling aspects of our case study was the potential cross-cultural applicability of combining CBT and SP.

There are several limitations to the findings of our study. From the psychodiagnostic perspective, the questionnaires used in this study were not validated in Bengali; therefore, they were translated by a professional cultural mediator trained in basic psychotherapeutic techniques to treat post-traumatic symptoms. This factor has interfered with the possibility of using a rigorous statistical method (i.e., Reliable Change index) to statistically validate the pre and post-psychodiagnostics outcomes. Therefore, the scores should be interpreted carefully, as the cross-cultural adaptation of a psychological self-administered questionnaire is a complex process. It requires not only accurate linguistic translation but also cultural adjustment relevant across diverse cultural contexts [110].

Other significant limits refer to the lack of control groups. Indeed, not comparing the client with subjects who did not receive any treatment weakens the generalizability of our results. Furthermore, not having comparisons with patients who received standardized interventions (i.e., CBT alone, SP alone, EMDR alone) prevents us from asserting our combined intervention effectiveness. Also, due to this, it is not possible to specify the contribution of each psychotherapy; in this sense, further studies are needed to define the efficacy of a single contribution.

In addition, a longer-term follow-up would be essential to verify the durability and stability of the observed changes through the therapy over time. Further research is needed to evaluate the effectiveness of this integrated approach by conducting multiple clinical case studies. These studies should assess its efficacy compared to other individual interventions and examine the specific contributions of each component (CBT and SP) to post-traumatic, dissociative, and depressive symptoms.

In conclusion, although any firm conclusion cannot be drawn because of various limitations that straiten our ability to generalize from these findings to other contexts, this case study represents the first attempt in which techniques taken from SP are used, integrated with other approaches and discussed systematically in a transcultural setting. Further research with larger samples across different countries is needed to validate these assumptions.

## Figures and Tables

**Table 1:** Scores on the PC-5, BDI-II and DES-II. The description highlighted in bold indicate the total score of each questionnaire.

Scales	Income	Outcome
**PCL-5 total**	61	24
Intrusion symptoms	19	5
Avoidance	8	3
Negative alterations in cognition and moods	19	9
Alterations in arousal	15	7
**DES-II Total**	**28.5**	**13.3**
Taxon	12.8	27.5
Depersonalization/Derealization	33.6	16.6
Amnestic dissociation	17.5	0
Absorption and imaginative involvement	36.6	12.2
**Hamilton-D**	**21**	**10**

**Table 2:** Emotional Part, somatic indicators, and cognitive beliefs.

Emotional Part (EP)	Somatic Indicators	Beliefs
Freeze	Feeling cold on the chest; increase in heart rate; dissociative states (“I can’t move the leg when lay down in bed”)	I am vulnerable I am not safe
Submit	“My legs are weak, flaccid”	I am powerless I can’t express my needs and my will, I have to indulge others’ desires
Flight	Rigidity of the posture	I am in danger I constantly avoid speaking with certain people [linked to the exploitation network]; If I would meet them, everything would start over. I have to lie
Attach for Survival part (relational level)	Body absent (as if it is not there) when interacting with others	I can’t trust anyone; Even if I can be with other people, I feel distant

## Data Availability

The data presented in this study are available on request from the corresponding author due to privacy.
